# Hydrogen storage performance of the multi-principal-component CoFeMnTiVZr alloy in electrochemical and gas–solid reactions

**DOI:** 10.1039/d0ra04089d

**Published:** 2020-06-29

**Authors:** Baran Sarac, Vladislav Zadorozhnyy, Elena Berdonosova, Yurii P. Ivanov, Semen Klyamkin, Selin Gumrukcu, A. Sezai Sarac, Artem Korol, Dmitri Semenov, Mikhail Zadorozhnyy, Adit Sharma, Alan L. Greer, Jürgen Eckert

**Affiliations:** Erich Schmid Institute of Materials Science, Austrian Academy of Sciences 8700 Leoben Austria; National University of Science and Technology “MISIS” Leninskyprosp., 4 119049 Moscow Russia zadorozhnyyvlad@gmail.com; Department of Chemistry, M. V. Lomonosov Moscow State University 119991 Moscow Russia; Department of Materials Science & Metallurgy, University of Cambridge Cambridge CB3 0FS UK; School of Natural Sciences, Far Eastern Federal University 690950 Vladivostok Russia; Department of Chemistry, Istanbul Technical University 34469 Istanbul Turkey; Polymer Science & Technology, Istanbul Technical University 34469 Istanbul Turkey; Department of Materials Science, Chair of Materials Physics, Montanuniversität Leoben 8700 Leoben Austria

## Abstract

The single-phase multi-principal-component CoFeMnTiVZr alloy was obtained by rapid solidification and examined by a combination of electrochemical methods and gas–solid reactions. X-ray diffraction and high-resolution transmission electron microscopy analyses reveal a hexagonal Laves-phase structure (type C14). Cyclic voltammetry and electrochemical impedance spectroscopy investigations in the hydrogen absorption/desorption region give insight into the absorption/desorption kinetics and the change in the desorption charge in terms of the applied potential. The thickness of the hydrogen absorption layer obtained by the electrochemical reaction is estimated by high-resolution transmission electron microscopy. The electrochemical hydrogen storage capacity for a given applied voltage is calculated from a series of chronoamperometry and cyclic voltammetry measurements. The selected alloy exhibits good stability for reversible hydrogen absorption and demonstrates a maximum hydrogen capacity of ∼1.9 wt% at room temperature. The amount of hydrogen absorbed in the gas–solid reaction reaches 1.7 wt% at 298 K and 5 MPa, evidencing a good correlation with the electrochemical results.

## Abstract 2

The single-phase multi-principal-component CoFeMnTiVZr alloy was obtained by rapid solidification and examined by a combination of electrochemical methods and gas–solid reactions.

## Graphical abstract

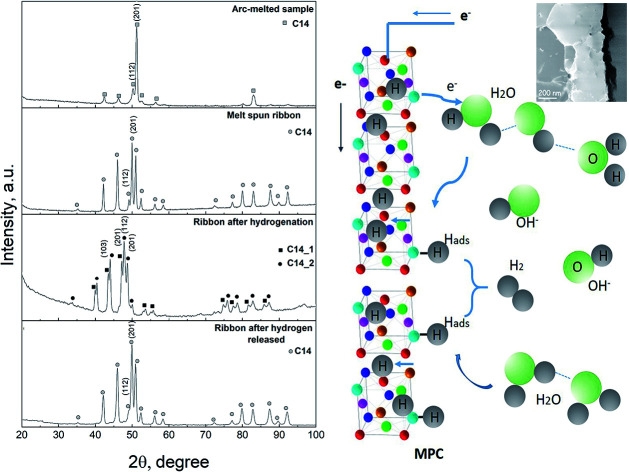

## 1 Introduction

Recently, multi-principal-component alloys (or high entropy alloys – HEAs) have attracted considerable attention. The interest in these alloys stems from their unique microstructure and unusual physical and mechanical properties, *i.e.*, high strength and hardness, exceptional wear and heat resistance, good structural stability, and high resistance to corrosion and oxidation.^1–7^ Multi-principal-component (MPC) alloys usually contain five or more main components, while the concentration of each element varies from 5 to 35 at%.^3,4^ Such alloys tend to form solid solutions with cubic body-centered (bcc) or cubic face-centered (fcc) crystal lattices.^8,9^ One of the promising applications of multi-principal-component alloys (MPCs) is the accumulation of hydrogen in the solid-state as a fuel for electricity and transport, *i.e.*, using these alloys for reversible hydrogen storage.^10^ The MPC alloys capable of accumulating hydrogen are promising for their ability to achieve a higher bulk density of hydrogen than in liquid hydrogen.^11–15^ For example, transition metals and their alloys are known to have high catalytic activity for hydrogen evolution reaction due to their partially filled d-orbitals mediating the gain and loss of electrons.^16^ To this extent, MPC alloys with a number of elements mixed in near equiatomic ratios offer promising properties as hydrogen-storage materials. The ability to crystallize in simple cubic structures in the presence of large lattice strain originates from the different sizes of the atoms by a two-stage hydrogen absorption reaction, starting with an intermediate monohydride and eventually forming a dihydride phase.^17^

The gas–solid reaction method or pressure-composition isotherm (PCI) measurements are widely used for the estimation of the reversible hydrogen storage capacity in different metallic alloys and intermetallic compounds.^18^ A considerable number of MPC alloy compositions have already been investigated by gas–solid reactions.^19–27^ However, in some cases, the estimation of the hydrogen-storage capacity by the gas–solid reaction method is impossible due to the low sensitivity of the PCI measurement (*e.g.*, for metallic-glass compositions). The estimate of the hydrogen capacity is also inaccurate for MPC alloys with different types of crystalline structures. Therefore, in such systems, the estimation of hydrogen storage by electrochemical reactions is more reasonable.^28–30^

The hexagonal C14 Laves phase structure is one of the promising types of crystalline structures for single-phase formation in MPC alloys; moreover, such type of structure is very promising for reversible hydrogen storage capacity. For example, Kopczyk *et al.*^31,32^ studied the MPC Zr–Ti–V–Ni–Cr–Fe alloy for different Ti and Zr ratios in alkaline electrolyte through potentiodynamic current overvoltage and galvanostatic over potential-time tests during long-term continuous and intermittent charge–discharge cycles. The pressure-composition isotherms for hydrogen absorption/desorption evaluated from the equilibrium potential were compared with the gas-phase isotherms.^32,33^ The kinetic data indicated the reversibility of hydrogen electrosorption in the investigated systems. It should also be noted that the electrochemical reaction of the reversible hydrogen storage investigation is very attractive for the analysis of metallic fillers used inside gas-separation composite membranes.^34^ To this extent, cyclic voltammetry (CV) is commonly implemented to investigate the redox (reduction and oxidation) processes and investigate the electron-transfer-based chemical reactions of elements and compounds.^35^ CV is also used to analyze the sorption and desorption behavior of hydrogen and electrocatalytic behavior in various materials and applications.^36–49^

Hydrogen absorption and evolution reactions have attracted growing interest in the past years due to their insufficient kinetics, *i.e.* low conductivity of OH^−^ ions in alkaline electrolytes.^50^ This slow kinetics may challenge the development of anionic exchange membrane water electrolyzers. For this reason, multi-component alloy systems can be envisioned to allow tuning of the ion transfer in the electrolytes and, thus, to enhance the absorption and electrocatalytic behavior. In contrast to the poor corrosion resistance of other MPC alloys, a Ni_20_Fe_20_Mo_10_Co_35_Cr_15_ MPC alloy with high corrosion resistance was reported as a highly active and stable electrocatalyst for the hydrogen evolution reaction (HER) in alkaline electrolytes.^16^ Moreover, it has been reported that at any given temperature, the Cu_0.5_NiAlCoCrFeSi MPC alloy has better resistance to general corrosion in 0.5 M H_2_SO_4_ than 304 stainless steel.^15^

MPCs and intermetallic compounds (IMC)^51–54^ modified by different alloying elements^55–59^ have been investigated as hydrogen-storage alloys in our previous research. In the present work, a combination of structural investigations, volumetric, electrochemical, and calorimetric experimental techniques is employed to study the hydrogen-storage features of the MPC CoFeMnTiVZr alloy obtained by rapid solidification (arc-melting with subsequent melt spinning). It should also be noted that the composition of the chosen MPC CoFeMnTiVZr alloy is much closer to compositions of new-generation hydrogen-storage alloys reported in the work of Cao *et al.*^60^ This similarity allows for a practical comparison of the alloy's hydrogen-storage properties in ref. 60 and the present alloy. Besides, the overall study of the methodology used in the present work is particularly interesting for assessing the applicability of electrochemical and physical methods for the evaluation of the hydrogen storage and release performance of novel materials.

## 2 Experimental details

### 2.1 Alloy preparation

Powders and chips of Co (purity 99.9%), Fe (purity 99.99%), Mn (purity 99.8%), Ti (purity 99.995%), V (purity 99.7%) and Zr (purity 99.95%) were used as starting components. Ingots of the Co–Fe–Mn–Ti–V–Zr alloy with an equiatomic composition were fabricated by arc melting (Edmund Bühler GmbH) of the metal mixture in an argon atmosphere purified by a Ti getter. Upon melting, the ingots were flipped over and re-melted five times to ensure compositional homogeneity. Ribbon samples were prepared by melt spinning in an argon atmosphere, where the ingot was melted by an induction coil and ejected by gas pressure on to a rotating copper wheel (Edmund Bühler GmbH). The thickness of the resulting ribbons varied from 20 μm to 30 μm, and the width ranged from 4.7 to 4.9 mm.

### 2.2 Analysis of the structure and phase composition

Analysis of the structure and phase composition of the obtained materials was carried out by a Bruker X-ray diffractometer (XRD) with Co Kα radiation. The determination of the crystal lattice parameters and the phase composition were provided with an accuracy of 0.0005 nm and 5%, respectively.^61^ The finite size of the coherent scattering domains (CSD) or crystallites in the obtained materials was determined by the approximation method from diffraction line broadening. A Cauchy function was used as an approximating function.^62^ In this case, CSD is a characteristic region of a crystal that scatters X-rays coherently and independently of other similar regions. Usually, the CSD size is used to estimate crystallite sizes in polycrystals or powder nanomaterials. In these cases, the CSD size is usually identified with the average crystallite size, although the real CSD size is smaller than the crystallite itself since it has an amorphous structure near the crystallite boundary.^63^

Transmission electron microscopy (TEM) studies were conducted using an FEI Tecnai Osiris with field-emission gun TEM/S (scanning) TEM operated at 200 keV, equipped with a Super-X windowless EDX detector. Cross-sectional specimens with a final thickness of 50–100 nm were prepared by focused ion beam (FIB) milling of the hydrogenated alloy using a Helios Nanolab FIB/SEM (scanning electron microscope).

### 2.3 Investigation of the hydrogen sorption properties by the gas–solid reaction method

To evaluate the hydrogen-absorption performance of the MPC alloy, pressure-composition isotherms (PCI) were measured using a handmade Sieverts type facility. The measuring system was connected to a Tian-Calvet DAK-1a calorimeter allowing direct determination of the reaction enthalpy during the interaction of alloys with hydrogen. In order to evaluate equilibrium and reproducible thermodynamic parameters, the samples were first activated in vacuum at 670 K and then subjected to three hydrogenation–dehydrogenation cycles.

To check the phase composition of the reaction product, the samples were quenched in liquid nitrogen under H_2_ pressure. Then the samples were exposed to air for 1–2 hours at the same temperature (77 K). This procedure leads to passivation of the sample surface and prevents hydrogen desorption for a few hours, allowing to perform XRD analysis.

### 2.4 Electrochemical measurements

The redox potential of Ag/AgCl (3 M NaCl) is +0.209 V *vs.* a reference hydrogen electrode (RHE) at 298 K. Samples were tested in an Ar-saturated 6 M KOH solution. The MPC alloy samples were saturated using chronoamperometry at given saturation potentials succeeded by CV measurements using a PARSTAT 4000A potentiostat (Princeton Applied Research, USA), with an applied current accuracy of 0.2% of the reading. The data points of the chronoamperometric saturation were taken in 1 second intervals. Cyclic voltammetry scan rates were selected as 20 mV s^−1^. The charge densities, the hydrogen-to-metal (H/M) ratio, and wt% of hydrogen at a given potential were calculated from *Q* = *A*_c_/*A*_s_ × ν. Here, *A*_c_ is the area under the desorption curve between the hydrogen evolution region (HER) and the double layer capacitance region, *A*_s_ is the surface area, and ν is the scan rate. The calculated density of the equiatomic CoFeMnTiVZr MPC is *ρ* = 6.604 g cm^−3^, the submerged surface area was *A* = 0.040 ± 0.004 cm^2^, and the effective thickness of the MPC exposed to OH^−^ ions was estimated from STEM high-angle annular dark-field imaging (HAADF) (from the average of 30 different thickness measurements) as 103 ± 2 nm.

## 3 Results and discussion

### 3.1 Alloy preparation, structure and phase composition analyses

**Fig. 1:**
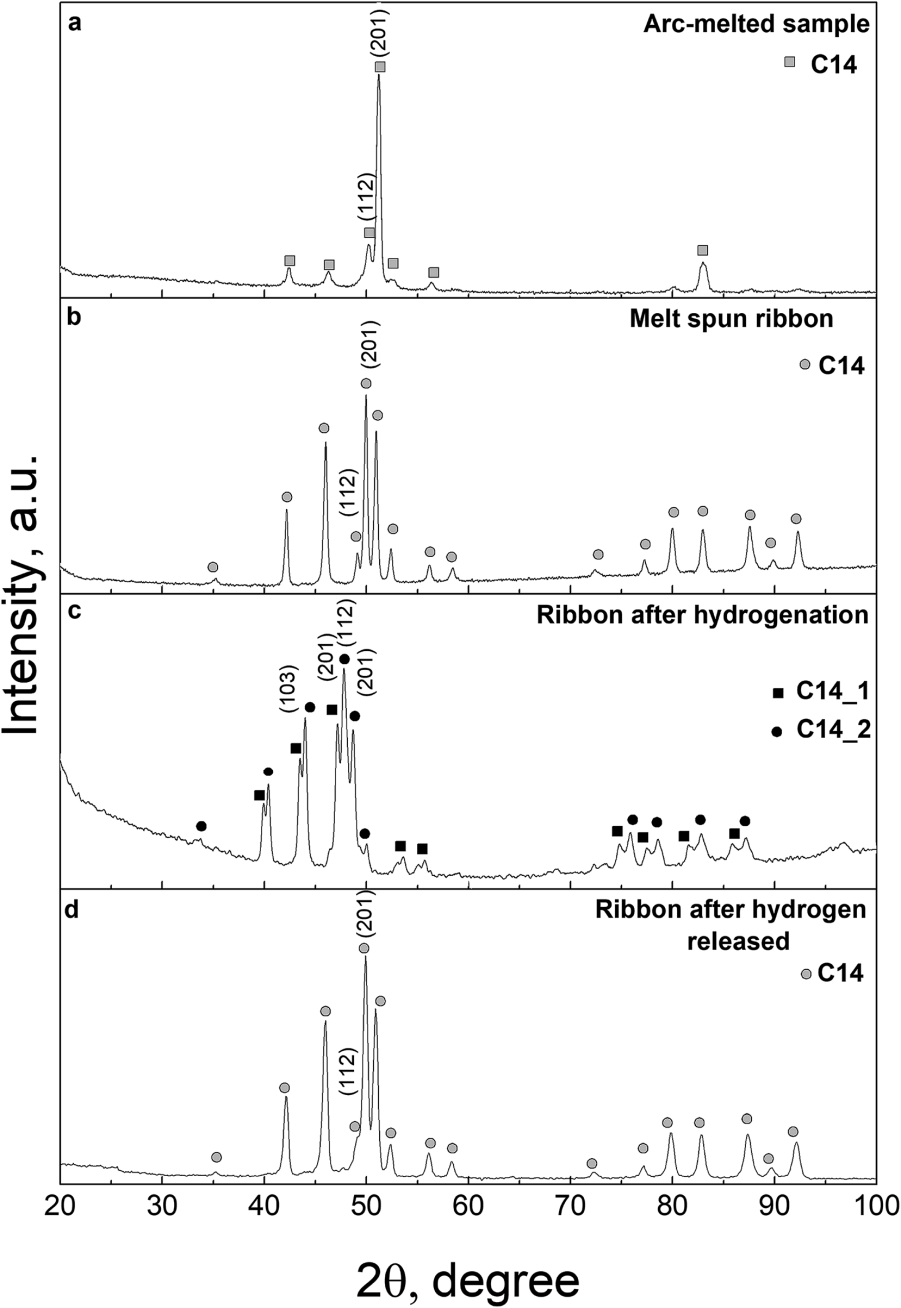
XRD patterns of the CoFeMnTiVZr MPC alloy: arc-melted sample (a), melt-spun ribbon (b), ribbon sample after hydrogenation (c), and subsequent hydrogen desorption (d).

The preparation of the single-phase equiatomic MPC CoFeMnTiVZr alloy with homogeneous grain structure is a complicated procedure in conventional arc-melting which results in texture orientation or formation of large grains. This orientation is clearly revealed by the significant integral intensity of the (201) line and the suppressed intensity of other reflections (Fig. 1a).

The XRD analysis of the as-cast alloy indicates the formation of a hexagonal Laves phase structure (C14 type) characteristic of intermetallic compounds formed by metals with a large difference in atomic radii as in the case of the ZrTiVNiCrFe MPC alloy.^51^ It is worth noting that there is a significant deviation of the relative intensity of the peaks in the XRD pattern from that in the powder diffraction pattern for this structure type (Fig. 1a). This deviation indicates a pronounced texture of the as-cast alloy and an inhomogeneous grain size distribution. The rapid solidification through melt spinning does not affect the lattice parameters but eliminates the above structural feature due to the specific 2D geometry of the ribbon (Fig. 1b) as compared with bulk samples (Fig. 1a).

XRD analysis of the hydrogenated sample evidences the formation of two hydride phases, both retaining the original C14 structure type, but differing in unit-cell parameters (Table 1). We have to emphasize the unusually low volumetric expansion of the crystal lattice generated by hydrogen absorption: for the synthesized hydrides, it is 8% and 13%, respectively. The formation of two hydride phases in the hydrogenated sample, together with the low volumetric expansion of the crystal lattice generated by hydrogen absorption, is in good agreement with previously reported data on the ZrTiVNiCrFe MPC alloy.^48^ Meanwhile, for most C14 type binary intermetallics, the volumetric expansion reaches and sometimes even exceeds 20% at the same hydrogen concentration.^18,64–68^

**Table 1:** Crystal structure parameters of the MPC CoFeMnTiVZr alloy and its hydrides

MPC CoFeMnTiVZr alloy	Lattice parameters, nm (C14 type)	CSD size (XRD observation), nm	Crystallite size (TEM observation), nm
Melt spinning (before hydrogenation)	*a* = 0.4972	≈20	≈400
	*c* = 0.8105		
After hydrogenation			
1st hydride			
Δ*V*/*V*_0_ ≈ 8%	*a* = 0.5105	≈40	
	*c* = 0.8344		
2nd hydride			
Δ*V*/*V*_0_ ≈ 13%	*a* = 0.5170	≈20	
	*c* = 0.8470		
After hydrogen released	*a* = 0.4976	≈30	
	*c* = 0.8101		

The significantly reduced effect of specific volume on hydride formation seems to be typical for MPC alloys. In essence, the record hydrogen absorption of H/M = 2.5 reported in ref. 10 for TiVZrNbHf leads to an increase of only 26%, *i.e.*, about 10% per 1 H/M. Such a feature is likely due to the lattice strain in the alloy caused by variations in atomic radii of the components.

Table 1 presents the structure parameters of the samples before and after hydrogenation. As can be seen from Table 1 and Fig. 1, after hydrogen desorption, the two-phase hydrogenated sample restores the single-phase structure of the as-produced alloy. We have an important finding that the hydrogen absorption/desorption process is reversible and proceeds without decay of the intermetallic matrix.

**Fig. 2:**
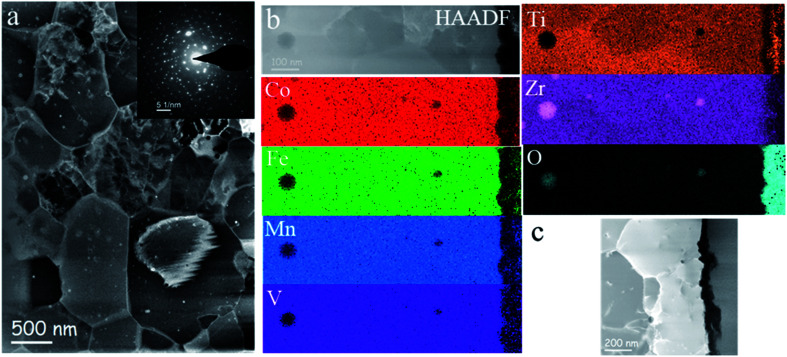
HAADF STEM image of the CoFeMnTiVZr–H_2_ alloy (a). A characteristic SAED pattern is shown in the inset, HAADF image and corresponding EDX maps (b), STEM DF image of a large region exposed to OH^−^ ions (black region) (c).

TEM investigations confirm a homogeneous submicrometer-grained microstructure of the CoFeMnTiVZr ribbons also after the electrochemical studies (Fig. 2a). The electron diffraction pattern shown in the inset indicates a hexagonal C14 type Laves phase structure with *a* = 0.497 ± 0.002 nm and *c* = 0.810 ± 0.002 nm. The slight texture in^2–12^ crystallographic direction is in agreement with the XRD data. The elemental mapping in Fig. 2b shows the compositional homogeneity of the sample. There are a few nanometer-sized zirconium oxide particles randomly distributed inside the alloy. The samples electrochemically saturated with hydrogen are characterized by the abundance of oxygen atoms originating from the hydroxyl ions, which do not show a significant modification of the microstructure and chemistry of the alloy. Recently, Lu *et al.* have observed corrosion behavior and passive film formation on the surface of Fe_50_Mn_30_Co_10_Cr_10_ Dual-Phase High-Entropy Alloy in sulfuric acid solution *via* XPS, where the Cr(OH)_3_ ions contributing to the passive oxide layer and the alloying elements were also indicated.^69^ Similar to their XPS findings, the dramatic changes observed for the oxygen content from our HAADF-EDX give us confidence that the passivation also takes place on the surface of our MPC alloy.

### 3.2 Gas–solid interaction in the CoFeMnTiVZr–H_2_ system

**Fig. 3:**
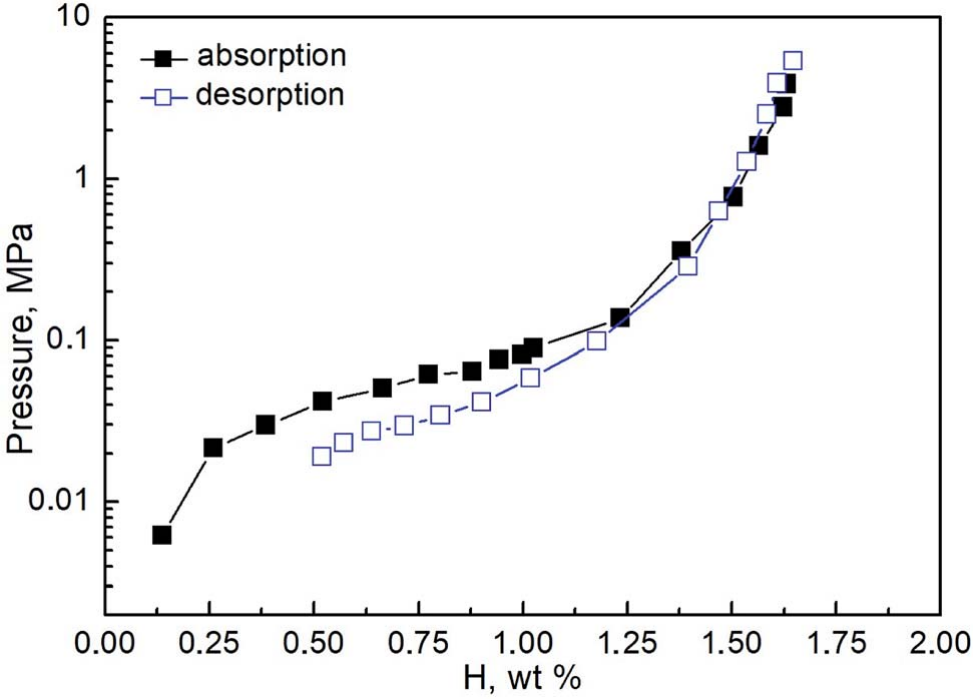
Pressure-composition isotherms in the CoFeMnTiVZr–H_2_ system at 298 K. Black filled squares – absorption; blue empty squares – desorption. The observational error of the measurements is of about 0.01–0.02 wt%.

The pressure-composition isotherms measured at room temperature for the studied system are shown in Fig. 3. The plateau at both absorption and desorption are rather sloped. That does not allow separating the regions corresponding to the formation of the two hydride phases. The maximum hydrogen absorption capacity reaches 1.7 wt%, correlating well with previously reported data.^60^ The low-pressure region on the desorption isotherm (below 0.01 MPa) could not be fully measured due to experimental limitations. To remove the residual hydrogen, the samples were degassed in a vacuum at 500 K between sequential absorption–desorption cycles. According to the calorimetric measurements, the hydrogenation enthalpy is evaluated as 36 ± 1 kJ mol^−1^ H_2_.

### 3.3 Electrochemical studies of the CoFeMnTiVZr alloy

**Fig. 4:**
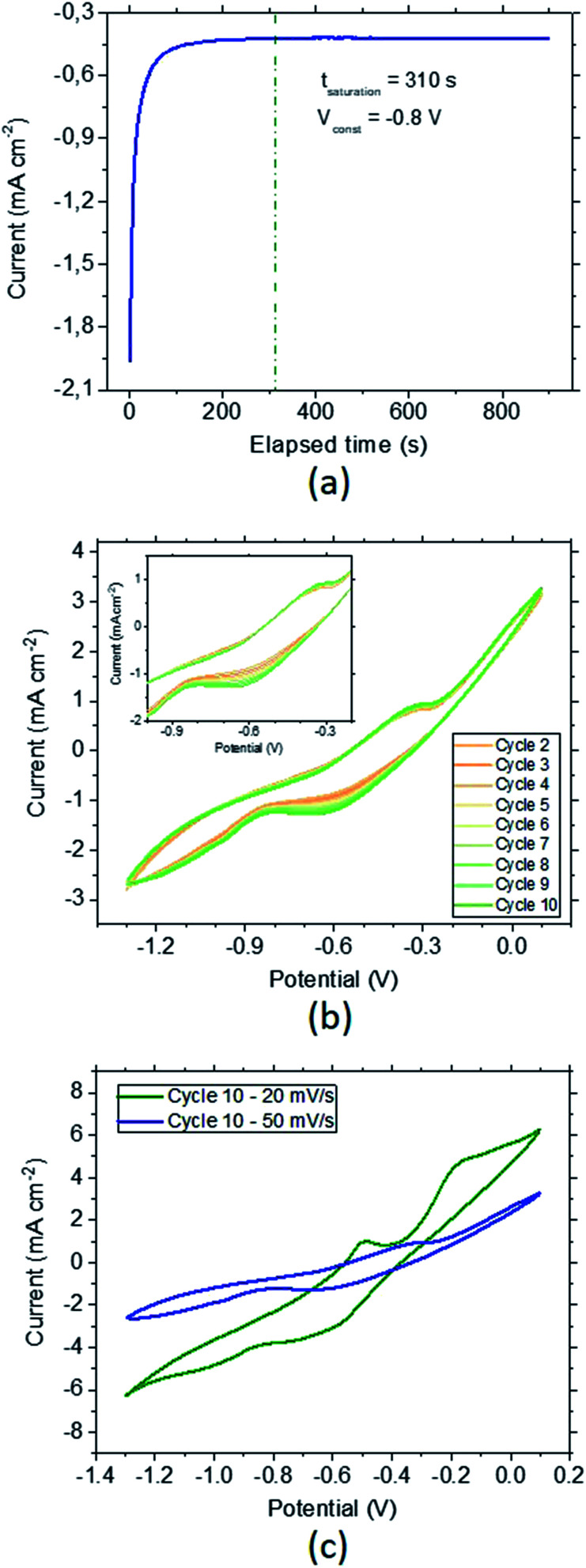
Hydrogenation of the MPC alloy by chronoamperometry. Full saturation was achieved by keeping the material at a constant potential of −0.8 V for more than 30 s in a 6 M KOH solution (a). The evolution behavior of the corresponding MPC alloy was subsequently analyzed by cycling the potential between −1.3 V and 0.1 V for 10 times at 50 mV s^−1^. The inset depicts the sorption (cathodic scan – below) and desorption (anodic scan – above) peaks. Note that the applied current is normalized by dividing it by the surface area of ∼0.04 cm^2^. The redox potential of Ag/AgCl (3 M NaCl) is +0.209 V *vs.* the normal hydrogen electrode at 298 K (b). Influence of scan rate on the hydrogenation/dehydrogenation kinetics (c).

Electrochemical hydrogenation of the CoFeMnTiVZr MPC alloy has been achieved by holding the material at constant voltage (chronoamperometry) (Fig. 4a). For this, an optimized potential of −0.8 V, which corresponds to the largest increase in the current due to hydrogenation followed by a constant current due to stabilized hydrogen saturation, was selected. Full saturation was established in ∼5 min. In order to check the material behavior after chronoamperometric saturation, cyclic voltammetry (CV) studies were conducted (Fig. 4b). The sorption peak appearing during the cathodic scan at −0.65 V, as well as the desorption peak appearing at the anodic scan at −0.35 V (Fig. 4b inset), stay constant after 10 cycles, confirming the stability of the fully hydrogenated samples. In order to understand the influence of the scan rate we performed the same test in a slower scan rate of 20 mV s^−1^. Fig. 4c depicts the 10^th^ CV cycle for the scan rates of 20 mV s^−1^ (green) and 50 mV s^−1^ (blue). Compared to 50 mV s^−1^, the desorption peak is more significant for the slower scan rates. Moreover, for a slower scan, a second desorption peak can be observed around −0.2 V.

A large separation was observed between the anodic and cathodic peaks. The features of these hydrogen signals can be strongly related to, *e.g.*, the bulk alloy composition and the effect of scan rate. Since the scan-rate effect does not remarkably differ, the effect of the sorption potential can be mainly related to the potential dependence on the bulk composition resulting in large changes between the anodic and cathodic signals.

**Fig. 5:**
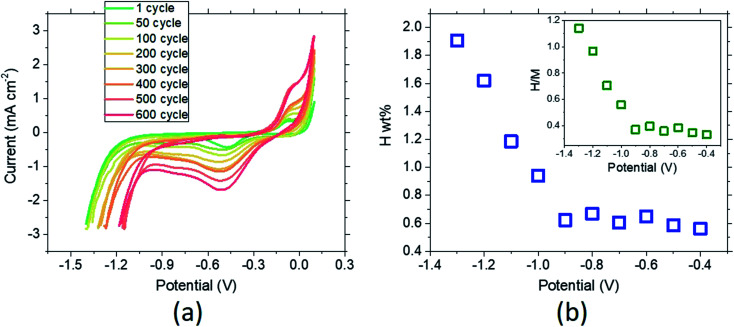
Long-term cyclic sorption and desorption behavior of the MPC alloy ribbons. The kinetics of hydrogen sorption is faster, resulting in the broadening of the sorption peak and a shift of the evolution region towards higher potentials. The applied scan rate is 100 mV s^−1^ (a). The change in the amount of hydrogen stored in the MPC alloy in terms of weight (b). A combinatorial chronoamperometry and CV approach was applied to calculate the charge of hydrogen calculated from the desorption region of each corresponding curve recorded at various potentials. The maximum hydrogen amount stored reaches ∼1.9 wt% at −1.3 V. Note that the applied current is normalized by dividing it by the surface area of ∼0.04 cm^2^. The redox potential of Ag/AgCl, 3 M (NaCl) is +0.209 V *vs.* normal hydrogen electrode at 298 K. The applied scan rate is 20 mV s^−1^. The H/M *vs.* applied potential is given in the inset. The error percent of the measurements lies within ∼10% of the recorded values (b).

The reversibility of the MPC alloy during hydrogenation is also an essential parameter for use in long-term hydrogen-conversion systems. Fig. 5a shows the CV curves of the material (unhydrogenated in the beginning) between −1.4 V and 0.1 V recorded at a scan rate of 100 mV s^−1^. The onset of the hydrogen evolution region (HER) determined from the initiation of the sharp current drop (∼−1.2 V) shifts to higher potential, which is accounted for by the increase of the stored hydrogen as the cycle time increases. The sorption peak at the cathodic part becomes very pronounced after 600 cycles (*c.f.* increase in the desorption peak at the anodic part). These results suggest that due to the difference in the sorption *vs.* desorption kinetics, the hydrogen storage becomes larger as the number of cycles increases. The desorption-to-sorption ratio (S/D) retrieved from the integration of the peaks results in S/D = 1.56 for the first cycle. This ratio greatly increases to S/D = 4.66 after 600 cycles, and the amount of total sorption becomes 7.33 times larger than in the initial state. Thus, another important finding is the durability of the selected MPC alloy after long-term reversible hydrogenation in the 6 M KOH electrolyte. In order to trace the amount of hydrogenation with respect to the applied potential, a combined study of chronoamperometry and CV was conducted (Fig. 5b). Starting from −0.4 V to −1.3 V, the MPC alloy was fully saturated at a constant potential, followed by a CV cycle. In the first hydrogenation cycle after chronoamperometry at −0.4 V, the material reaches 0.56 wt%, where the stored hydrogen content slightly increases as the potential decreases. A sharp increase is observed at −1.0 V, where maximum hydrogenation is achieved at −1.3 V by 1.91 ± 0.20 wt% hydrogen sorption. Further decrease in the potential leads to stagnation and a drop in the hydrogen amount due to dominating hydrogen evolution. When the results are given in mole percentages (Fig. 5b inset), the maximum hydrogen-to-metal ratio, (H/M)_max_ = 1.14, is smaller than is found from the weight percent.

The main distinction between alkaline and acidic media is that the concentration of protons is significantly decreased in an alkaline environment. Thus, the Volmer and Heyrovsky steps are expected to include a water-dissociation step,^70,71^ as shown below:H_2_O + e^−^ + MPC = MPC-H + OH^−^ (Volmer)H_2_O + e^−^ + MPC-H = H_2_ + OH^−^ (Heyrovsky)

**Fig. 6:**
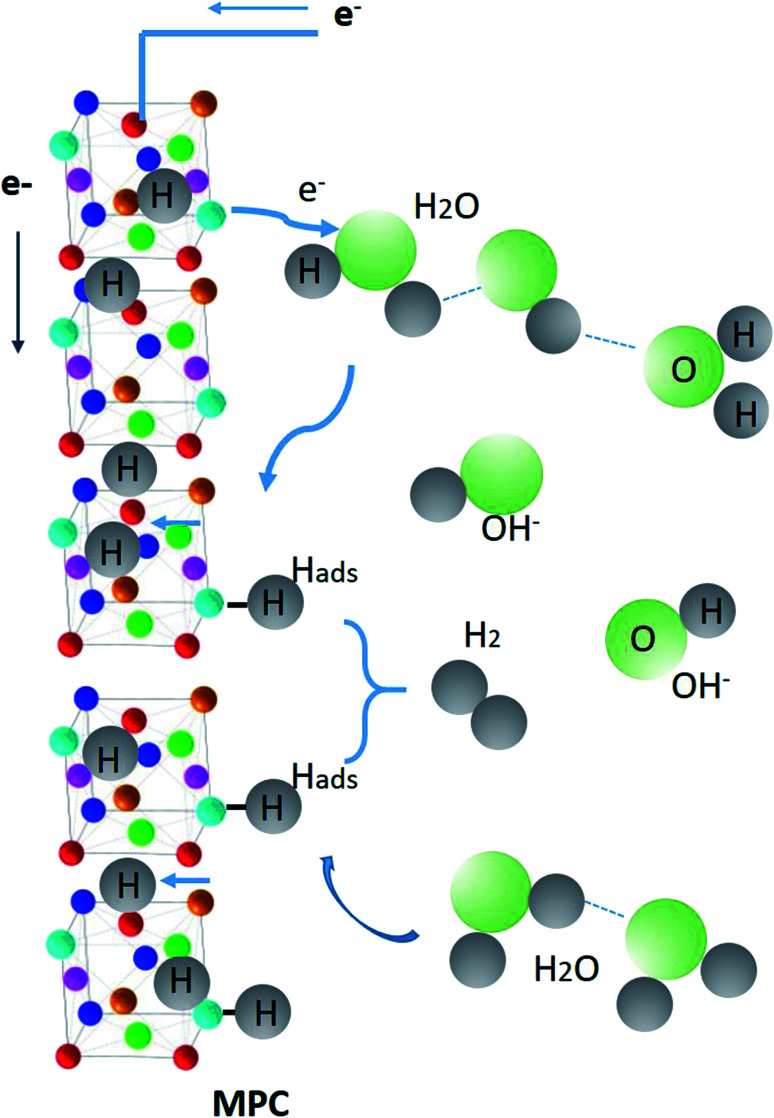
Schematics of the adsorption and absorption of hydrogen and release of hydroxyl ions and hydrogen to and from the MPC alloy ribbons. H_2_ and OH^−^ are shown larger than the size of MPS alloy components for convenience.

As reported in ref. 72, the hydrogenation kinetics over Pt (111) under alkaline conditions improved by the presence of OH^−^ ions, *i.e.*, defects and hydroxyl groups on the catalyst surface, can also enhance the H_2_O dissociation. In the case of CoFeMnTiVZr MPC alloy, the tendency to form oxide from the OH^−^ ions *via* hydrolysis (as confirmed by the high presence of the oxygen atoms in the form of oxides from the STEM-EDX measurements) is possible. The affinity of oxophilic groups to OH_ads_ is desired to be either strong (surface poisoning) or weak (no binding) to advance the hydrogen sorption/desorption and HER kinetics. The large lattice strain in this alloy makes it favorable to absorb hydrogen in both tetrahedral and octahedral interstitial sites.^10^ Thus, hydrogenation is confirmed by the presence of OH^−^ ions coming from the electrolysis of water in the alkaline aqueous solution. Fig. 6 schematizes the adsorption and absorption of hydrogen and the release of hydroxyl ions and hydrogen to and from the MPC alloy ribbons.

The hydrogen diffusivity (*D*) was estimated from the Cottrell eqn (1) provided in:^73^1
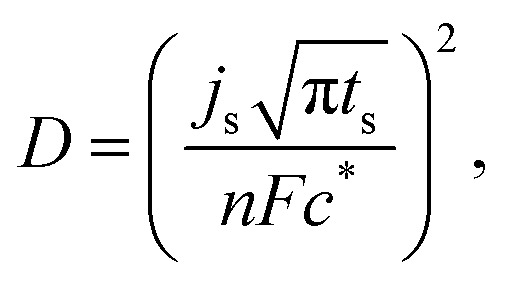
where *j*_s_ (A cm^−2^) is the current density at the time of saturation, *t*_s_(s) is the time for saturation, *n* is the number of electrons (taken here as 1), *F* is the Faraday constant (96 485 C mol^−1^), and c* is the initial concentration of the electrolyte (6 M KOH). Hence, the equation yields *D* = 5.27 × 10^−14^ m^2^ s^−1^. This value is very reasonable when compared to the hydrogen diffusivity of a similar MPC type alloy – Fe_20_Mn_20_Ni_20_Co_20_Cr_20_, on the order of 10^−11^ m^2^ s^−1^ hydrogenated by the same chronoamperometric method at 573 K.^74^

## 4 Conclusions

The single-phase multi-principal-component CoFeMnTiVZr alloy with C14 type Laves phase structure was prepared by rapid solidification. As established using the gas–solid reaction method (pressure composition isotherms measurements), the alloy has good stability in the reversible hydrogen absorption–desorption process, and its capacity reaches 1.7 wt% at room temperature. The electrochemical investigations demonstrate a gradual increase in the amount of absorbed hydrogen up to 1.9 wt% after 600 cycles. The obtained results verify that the electrochemical methods are very helpful for defining the hydrogen sorption/desorption kinetics and the corresponding amount of weight percent of stored hydrogen at each potential interval. The performed study demonstrates the efficiency of the employed electrochemical technique as an alternative to the conventional gas–solid reaction method, particularly for alloys with a low rate of desorption.

## Conflicts of interest

There are no conflicts to declare.

## Supplementary Material
